# Determinants for a Successful Sémont Maneuver: An *In vitro* Study with a Semicircular Canal Model

**DOI:** 10.3389/fneur.2016.00150

**Published:** 2016-09-16

**Authors:** Dominik Obrist, Andrea Nienhaus, Ewa Zamaro, Roger Kalla, Georgios Mantokoudis, Michael Strupp

**Affiliations:** ^1^ARTORG Center for Biomedical Engineering Research, University of Bern, Bern, Switzerland; ^2^Department of Otorhinolaryngology, Head and Neck Surgery, University Hospital Bern, Inselspital, Bern, Switzerland; ^3^Department of Neurology, Division of Cognitive and Restorative Neurology, University Hospital Bern, Inselspital, Bern, Switzerland; ^4^Department of Neurology, German Center for Vertigo and Balance Disorders, University Hospital Munich, Munich, Germany

**Keywords:** vertigo, BPPV, Sémont liberatory maneuver, canalolithiasis, semicircular canal

## Abstract

**Objective:**

To evaluate the effect of time between the movements/steps, angle of body movements as well as the angular velocity of the maneuvers in an *in vitro* model of a semicircular canal (SCC) to improve the efficacy of the Sémont maneuver (SM) in benign paroxysmal positional vertigo.

**Materials and Methods:**

Sémont maneuvers were performed on an *in vitro* SCC model. Otoconia trajectories were captured by a video camera. The effects of time between the movements, angles of motion (0°, 10°, 20°, and 30° below the horizontal line), different angular velocities (90, 135, 180°/s), and otoconia size (36 and 50 μm) on the final position of the otoconia in the SCC were tested.

**Results:**

Without extension of the movements beyond the horizontal, the *in vitro* experiments (with particles corresponding to 50 μm diameter) did not yield successful canalith repositioning. If the movements were extended by 20° beyond the horizontal position, SM were successful with resting times of at least 16 s. For larger extension angles, the required time decreased. However, for smaller particles (36 μm), the required time doubled. The angular maneuver velocity (tested between 90 and 180°/s) did not have a major impact on the final position of the otoconia.

**Interpretation:**

The two primary determinants for success of the SM are the time between the movements and the extension of the movements beyond the horizontal. The time between the movements should be at least 45 s. Angles of 20° or more below horizontal line (so-called Sémont+) should increase the success rate of SM.

## Introduction

Vertigo and dizziness are two of the most frequent symptoms occurring in neurology departments, with a lifetime prevalence of about 30% (1, 2). Their most common cause is benign paroxysmal positional vertigo (BPPV). BPPV is reported to have a prevalence between 10.7 and 64.0 cases per 100,000 population and a lifetime prevalence of 2.4% (3–5). Sémont’s liberatory maneuver (SM) (6) is an established method for treating BPPV due to canalolithiasis of a posterior semicircular canal (SCC) (7, 8). The original version was simplified and now consists of a two-step change in body position to flush otolithic debris out of the SCCs and back into the utricle (6, 9). The patient is seated sideways on an examination couch and is moved rapidly from the sitting position to the lying position with the head turned 45° opposite to the direction of movement. Sémont et al. (6) suggested that the patient remains for 2–3 min in this position. Then, the patient is rapidly moved in a 180° cartwheel motion to the opposite side without pause, while the head remains turned by 45°. The patient is brought into a seated position after resting for 5 min (6). Such a long time, however, is not generally recommended anymore (2, 10).

Although some studies indicate an effectiveness of SM of over 70% (11) and of 80–90% (12), clinical experience shows that the SM has to be repeated several times, BPPV remains unresolved in some cases, or it is subject to recurrences within a short time. Lee et al. (13) report an effectiveness of SM of less than 40% after the first maneuver. Moreover, it is “unclear what strategy should be pursued if the initial maneuver is not effective” (14), and a success rate of only 58% is reported for self-administered SM (4), which underlines the importance of effective teaching of repositioning maneuvers (15). Different recommendations have been made in clinical practice as to how to perform the SM with regards to the time between the different steps and the angular velocity of the body movements: recommended times between the two movements range from 30 s to 5 min (6, 16, 17); according to Sémont et al. (6), the movements should be performed quickly (without quantitative specification of velocity), while others suggested high acceleration with a maximal time duration of 1.5 s for the second movement by 180° (18). None of these recommendations were tested.

For the present study, we take the position that BPPV is due to canalolithiasis, i.e., due to free-floating particles in the SCC (19). An intraoperative study with patients undergoing labyrinthine surgery related these particles to degenerated otoconia and showed a significant association between such particles and BPPV (20). Here, we are excluding the case of cupulolithiasis, which is caused by cupular deposits (21) that alter the biomechanics of the cupula leading to positional nystagmus (22). Experiments with bullfrog labyrinths also indicated that canalolithiasis is more likely the cause for BPPV than cupulolithiasis (23). This was confirmed by theoretical work indicating that the nystagmus expected due to canalolithiasis is more consistent with clinical observations than a nystagmus due to cupulolithiasis (22).

**Figure 1:**
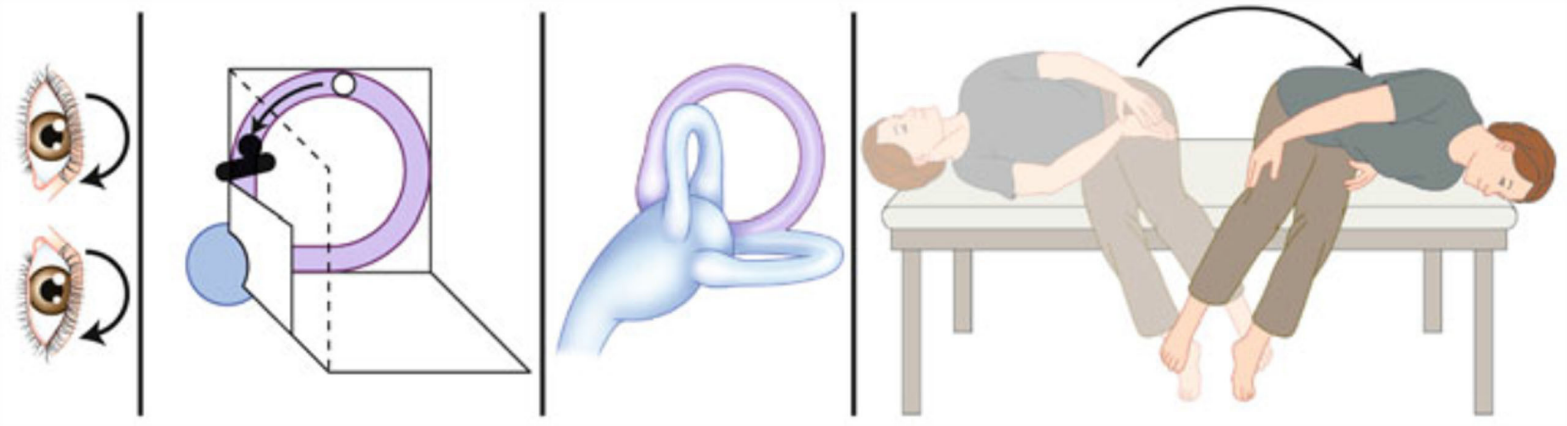
**Unsuccessful Sémont maneuver**. Schematic drawing of an unsuccessful Sémont maneuver. After the patient with right-sided BPPV is moved by 180° from right to the left, the particles do not move ampullofugally but ampullopetally into the direction of the cupula. This causes an ampullopetal cupula deflection with a reversed positional nystagmus with the quick phase beating to the left and downward. It indicates that the liberatory maneuver failed and must be repeated [adapted from Brandt et al. (8)].

Theoretically, it is ideal if the otoconia reach the lowest point relative to gravity in the SCC after each step of the SM to reduce the risk that they move toward the cupula, which would result in a failure of the maneuver (8) (Figure 1).

Therefore, we evaluated the different parameters that could have an effect on the movement of the otoconia and on their final position during the SM and thereby on the efficacy of the SM: first, the angular velocity during the maneuver; second, the resting time after the first movement of the patient; and third, the range of movement. We performed experiments with an *in vitro* model of an SCC (SCC model) with canaliths (24) to obtain quantitative evidence, relating the success of the SM to these therapeutic parameters. On the basis of these findings, we formulate clinical recommendations to increase the success rate of the SM.

## Materials and Methods

Two sets of *in vitro* experiments were conducted in this study. In the first set, the SCC model was mounted on a computer-controlled stepper motor, which performed parametrized SMs in a repeatable fashion. The center of rotation was at the center of the SCC model. In the second set of experiments, the center of rotation differed from that of the SCC model, mimicking a patient pivoting about the pelvis. To this end, the SCC model was mounted on a lever-arm device operated by an experienced neuro-otologist.

### Parametrized Sémont Maneuver

**Figure 2:**
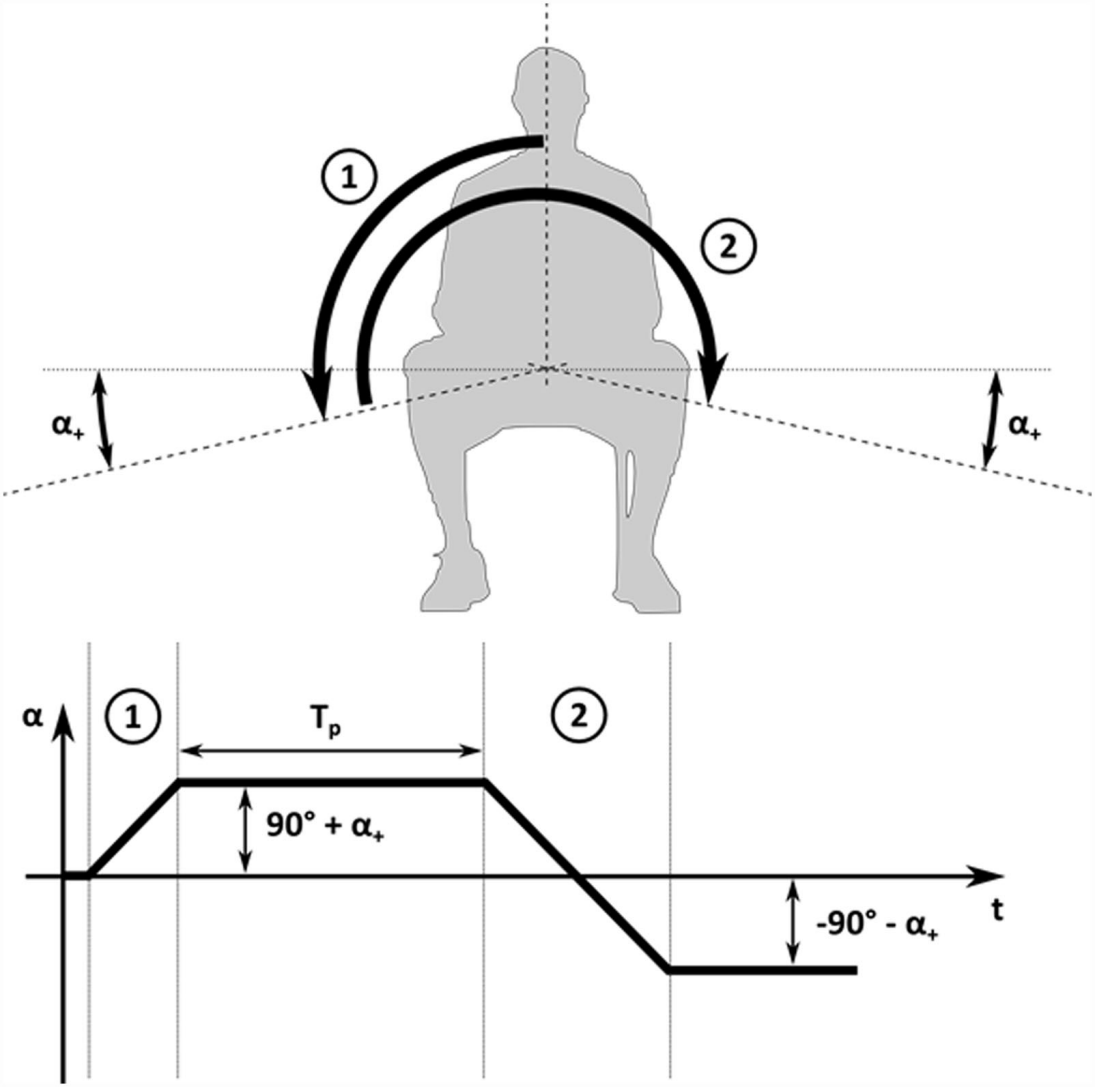
**Schematic drawing of parametrized Sémont maneuver**. Parametrized SM with first movement to the right (1), resting time *T*_P_, second movement to the left (2), and a final resting period on the left side.

The parametrized SM (Figure 2) used in the present study consisted of four phases: (1) first movement of the patient to the right by an angle of 90° + α_+_; (2) resting period of *T*_p_ seconds while the patient lies on the right side; (3) second movement of the patient to the left by 180° + 2α_+_ so that the SCC comes to rest on the left side at an angle of 90° + α_+_; and (4) final waiting period and assessment of the success of the maneuver.

This maneuver is defined by three parameters: the angular maneuver velocity $\dot{\text{α}}\left[{}^{\circ}/\text{s}\right]$, which defines the speed for the two movements; the resting time *T*_p_ [s] between the first and second step; and the extension angle α_+_ [°], which defines how much the SCC is rotated beyond the horizontal position. This extended movement range between the different body positions can be attained by an additional tilting of the head beyond the horizontal as confirmed by SMs performed on test persons.

### *In Vitro* Model for SCC with Canalolithiasis

**Figure 3:**
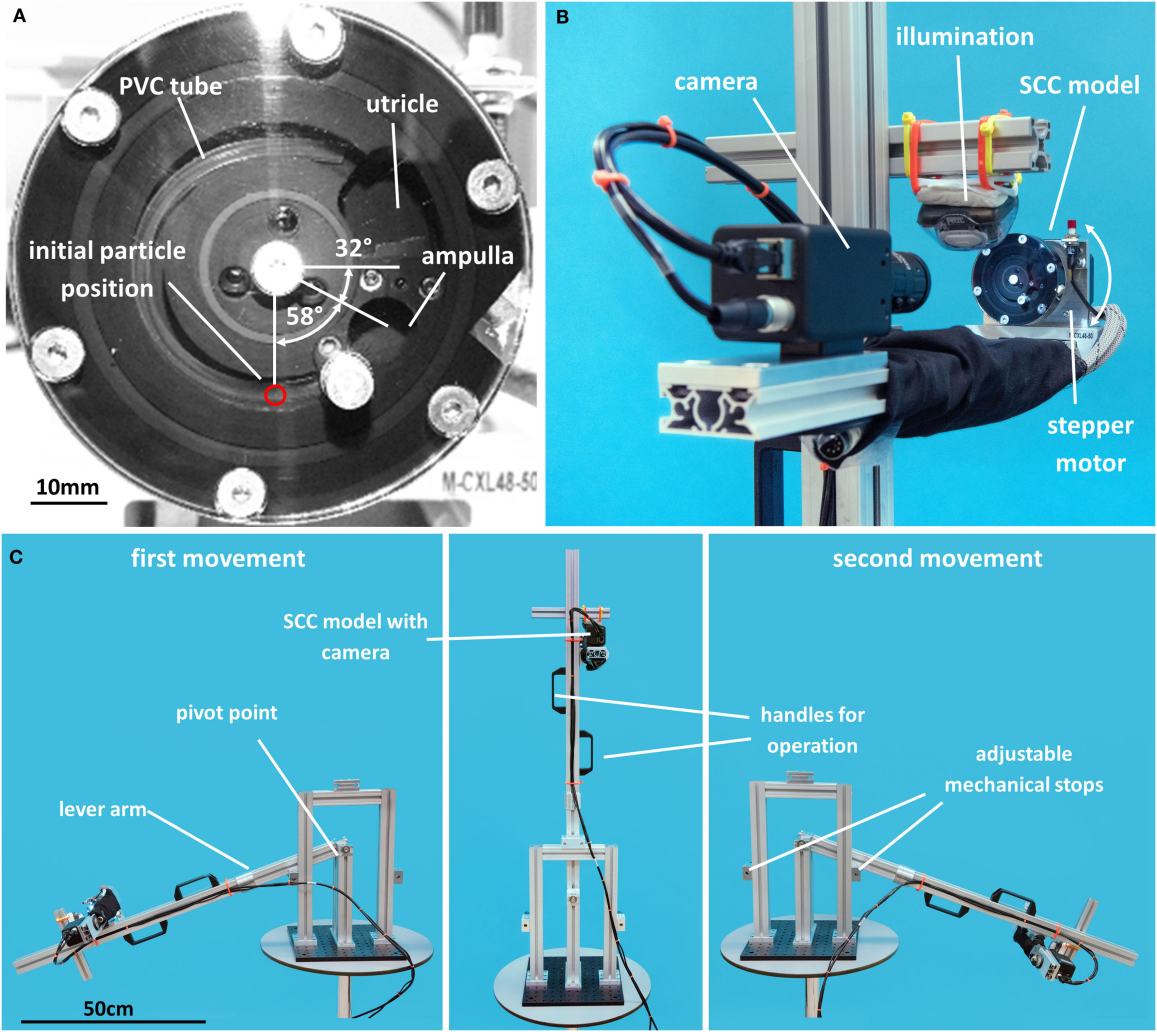
**Experimental setup**. **(A)** Scaled SCC model; **(B)** experimental setup with stepper motor; **(C)** setup for the lever-arm experiments.

A scaled SCC model (24) was used to analyze the behavior of otoconia. For simplicity, the SCC model was limited to the membranous duct of a single SCC; the ampulla and the utricle were filled with a viscous fluid modeling the endolymph (Figure 3A). The two other SCCs, the saccule and the bony labyrinth were omitted. Small particles were added to model otoconia. For better handling and accessibility, the SCC model and the particles were five times larger than in a human inner ear. The slender membranous duct of the SCC was modeled by a PVC tube (LabMarket, Ludwigshafen, Germany) with an inner diameter of 1.5 mm. This tube opened at one end to the utricle. The other end connected to the ampulla, which was closed by a 50-μm elastic membrane (Goodfellow Cambridge, Huntingdon, UK) modeling the cupula. We used Glycerintricapprylat (Blaser Swisslube, Hasle-Rüegsau, Switzerland) as a model for the endolymph. The otoconia were modeled by steel microspheres (MPS Micro Precision Systems, Biel, Switzerland) made of E52100 steel with diameters *D* of 180 and 250 μm. The material parameters (e.g., fluid viscosity, fluid density, particle density) were chosen so that the dynamic behavior of the SCC model was comparable to a human SCC with otoconia. For the geometrical scaling factor *f* = 5, the correct physical scaling (24) required, for example, that the fluid viscosity was *f*^2^ = 25 times larger than the viscosity of endolymph. For the particle density, the physical scaling requires that the ratio between particle and fluid density is 1 + *f* ⋅ (ρ_p_/ρ − 1) = 9.5 (where ρ_p_/ρ = 2.7 is the ratio of otoconia and endolymph density).

Table 1 summarizes the geometrical and physical properties of the scaled SCC model. The last column of Table 1 shows the values for a corresponding unscaled SCC with a major radius of 3.2 mm and a slender duct radius of 0.15 mm, which compares well to measurements of human SCC (25). The unscaled fluid density was 8% higher than the endolymph viscosity of 10^−6^ m^2^/s (26), and the unscaled particle density was about 9% lower than the density of otoconia (2700 kg/m^3^) (26). We can further quantify this difference by computing the particle settling time scale (24), which describes a characteristic time required by a canalith to fall through a short section of the SCC. It amounted to 1.17 s in the SCC model and to 0.92 s in a corresponding human SCC. Therefore, the particles in the SCC model settled about 20% more slowly than in a corresponding human SCC.

**Table 1:** **Geometrical and physical properties of the SCC model together with the corresponding unscaled parameters assuming a geometrical scaling factor of 5**.

		SCC model	Unscaled SCC
SCC radius (mm)	*R*	16	3.2
Radius of slender duct (mm)	*a*	0.75	0.15
Fluid viscosity (m^2^/s)	*ν*	27 × 10^−6^	1.08 × 10^−6^
Fluid density (kg/m^3^)	ρ_e_	945	1000
Particle diameter (μm)	*D*	180, 250	36, 50
Particle density (kg/m^3^)	ρ_p_	7800	2450

For the validation of this model, a 120° maneuver of the SCC model with particles has been studied (24). Particle motion (settling time and velocity) as well as cupula displacements in the *in vitro* model were shown to be comparable to the positional nystagmus in BPPV patients.

### Experimental Setup with Stepper Motor

For the first experimental setup, the SCC model was mounted on a stepper motor, which rotates the model (Figure 3B). The SCC model was aligned with the plane of rotation to mimic the turning of the head by 45°. The stepper motor was controlled by a personal computer to perform parametrized SMs. The movement of the particles was recorded with a video camera (JAI RM-6740 GE, Stemmer Imaging, Pfäffikon, Switzerland) and a 16-mm MegaPixel fixed FL lens (Edmund Optics, Karlsruhe, Germany). The recorded videos were analyzed with MATLAB (MathWorks, Natick, MA, USA).

### Lever-Arm Setup for Manual Maneuver

For the second experimental setup, the SCC model was mounted on a lever-arm device (Figure 3C) that could be freely rotated to the left and right. Adjustable mechanical stops on the left and right sides limited the range of motion of the lever arm to ±(90° + α_+_). The camera was fixed on the lever arm so that it moved with the SCC model. The length of the lever arm was 72 cm, thus corresponding to a typical distance from the pelvis to the SCC in average European men (DIN 33 402-2:2007). Two handles attached to the lever arm allowed an experienced neuro-otologist to perform the parametrized SM. A stopwatch was used to perform the SM with the given velocity and resting times. In addition, the SM was monitored electronically by attaching accelerometers (EyeSeeCam, Fürstenfeldbruck, Germany) to the SCC model.

### Experimental Protocol

Experiments with the stepper-motor setup and with the lever-arm setup were performed for a range of different parameters for the SM and for different particle sizes (Table 2). For simplicity, only one particle was used. We varied the extension angle α_+_ between 0° and 30° in steps of 10°, and the tested maneuver velocities α_+_ were 90, 135, and 180°/s. These parameter ranges were determined to be relevant for clinical maneuvers based on *ad hoc* measurements of several SMs using video-oculography goggles with accelerometers (EyeSeeCam, Fürstenfeldbruck, Germany).

**Table 2:** **Experimental configurations (particle diameter *D*, extension angle α_+_, maneuver velocity *v*) and the corresponding critical resting times (printed in bold) determined by stepper-motor and by lever-arm experiments (“X” indicates that a successful SM could not be performed for the tested range of resting times)**.

Particle diameter *D*	Extension angle α_+_	Maneuver velocity *v*		
		90°/s	135°/s	180°/s
**Stepper-motor experiments**				
180 μm	20°	**29 s**	**26 s**	**24 s**
250 μm	0°	**X**	**X**	**X**
250 μm	10°	**X**	**33 s**	**26 s**
250 μm	20°	**16 s**	**13 s**	**12 s**
250 μm	30°	**9 s**	**8 s**	**8 s**
**Lever-arm experiments**				
250 μm	20°	**16 s (12 s)**	**11 s (8 s)**	**10 s (8 s)**

*For the lever-arm experiments, the lower values in parentheses indicate the critical resting times for which SMs were only intermittently successful*.

At the beginning of each experiment, the SCC model was positioned such that the angle between the horizontal and the ampulla amounted to 32° (Figure 3A). This corresponds to the SCC orientation in upright position (27, 28). Before starting the experiment, it was ensured that the PVC tube was free of air bubbles and that the particle had settled at the lowest position in the SCC, i.e., 58° away from the ampulla.

After the second movement of the SCC model, the SM was considered successful if the particle settled in the utricle or unsuccessful if it settled in the ampulla (cf. Figure 3A).

For a given maneuver velocity, extension angle, and particle diameter, the resting time *T*_p_ was slowly increased from experiment to experiment in increments of 1 s or more (starting with a resting time of 5 s). This procedure was continued until a critical time could be identified, which marked the border between unsuccessful and successful repositioning, i.e., the critical time indicates the shortest possible resting time after which particles can be successfully repositioned.

For configurations close to the critical time, the results from the lever-arm experiments showed some variability, i.e., subsequent instances of the same experiment did not always yield the same results. Therefore, each experiment was repeated several times, and a maneuver was considered successful or unsuccessful only if at least three consecutive repetitions of that maneuver yielded successful (or unsuccessful, respectively) repositioning of the canalith. The stepper-motor experiments exhibited a high repeatability due to the computer-controlled maneuver. Nevertheless, the stepper-motor experiments were also repeated close to the critical time to ensure that external factors (e.g., room temperature, vibrations) did not affect the results.

## Results

### Canalith Trajectories

**Figure 4:**
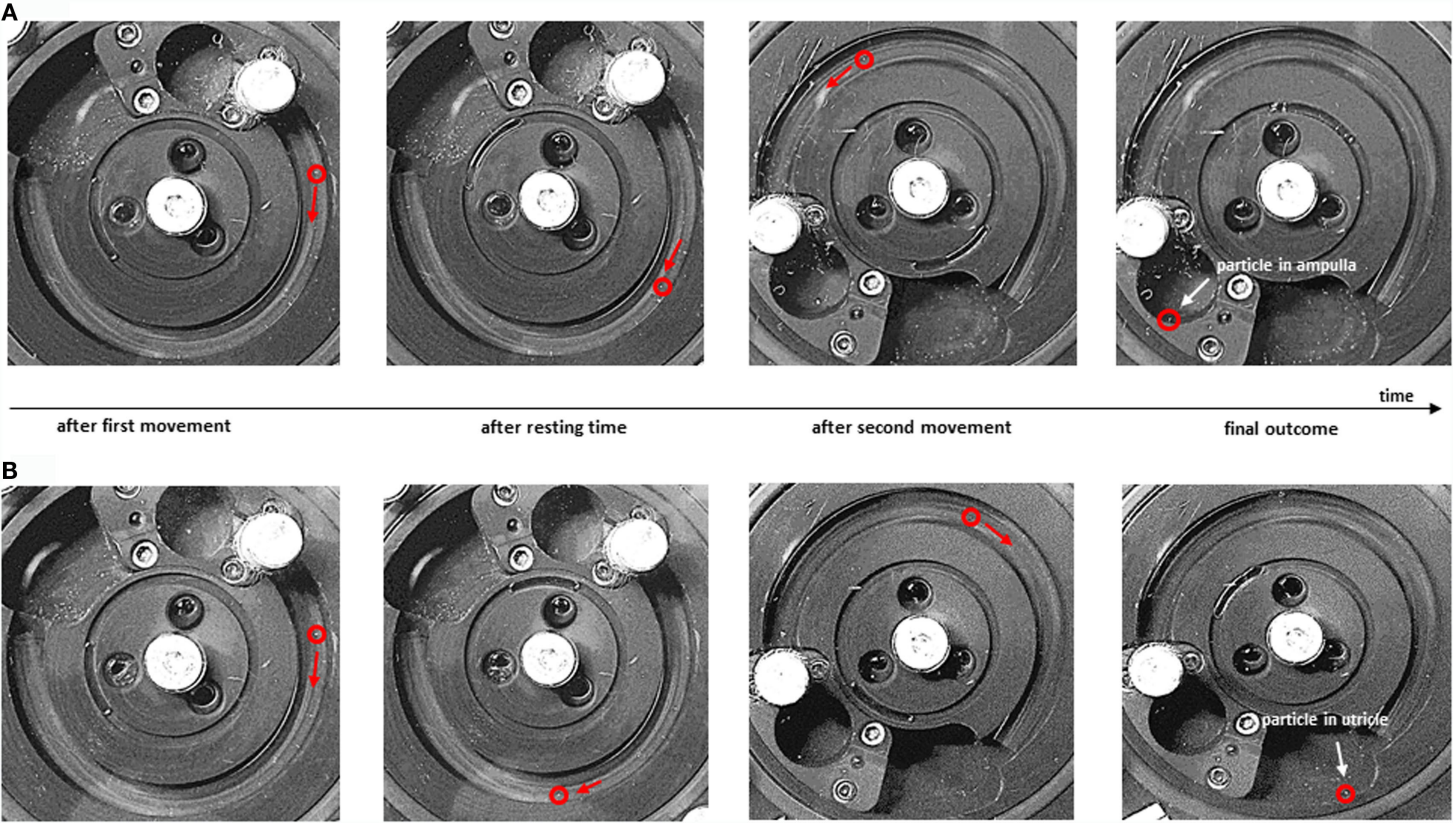
**Particle trajectories during Sémont maneuver**. Particle positions at different points in time during the SM (*D* = 250 μm, α_+_ = 20°). **(A)** Unsuccessful maneuver (*T_p_* = 5 s, *v* = 135°/s); **(B)** successful maneuver (*T_p_* = 45 s, *v* = 135°/s). From left to right: particle position (red circle) at the end of the first movement, after the resting time, after the second movement, and after the final resting time when the particle has settled **(A)** in the ampulla or **(B)** in the utricle of the SCC model.

Figure 4 illustrates the trajectory of a canalith in the SCC model, showing the position (and direction of motion) of the particle in stepper-motor experiments at different points in time for two exemplary SMs (also see the Video S1 in Supplementary Material). Figure 4A shows an unsuccessful repositioning (*Tp* = 5 s, *v* = 135°/s). The resting time *Tp* after the first movement is too short; the particle does not have enough time to settle at the lowest point of the SCC before the second movement begins. Consequently, the particle is not moved beyond the apex by the second movement, and it falls into the ampulla (*unsuccessful SM*). In the SM in Figure 4B (*Tp* = 45 s, *v* = 135°/s), the resting time is longer; the particle has time to settle at the lowest point of the SCC after the first movement. Therefore, the particle in Figure 4B is moved beyond the apex by the second movement, and it falls into the utricle of the SCC model (*successful SM*).

### Variation of Extension Angle

A set of experiments was performed with the stepper-motor setup using a particle with a diameter of 250 μm, different extension angles α_+_, and different maneuver velocities. For α_+_ = 0°, all attempts to successfully reposition the canaliths failed. Even in experiments with non-physiologically high velocities and/or extensively long resting times, it was not possible to reposition the particle into the utricle.

**Figure 5:**
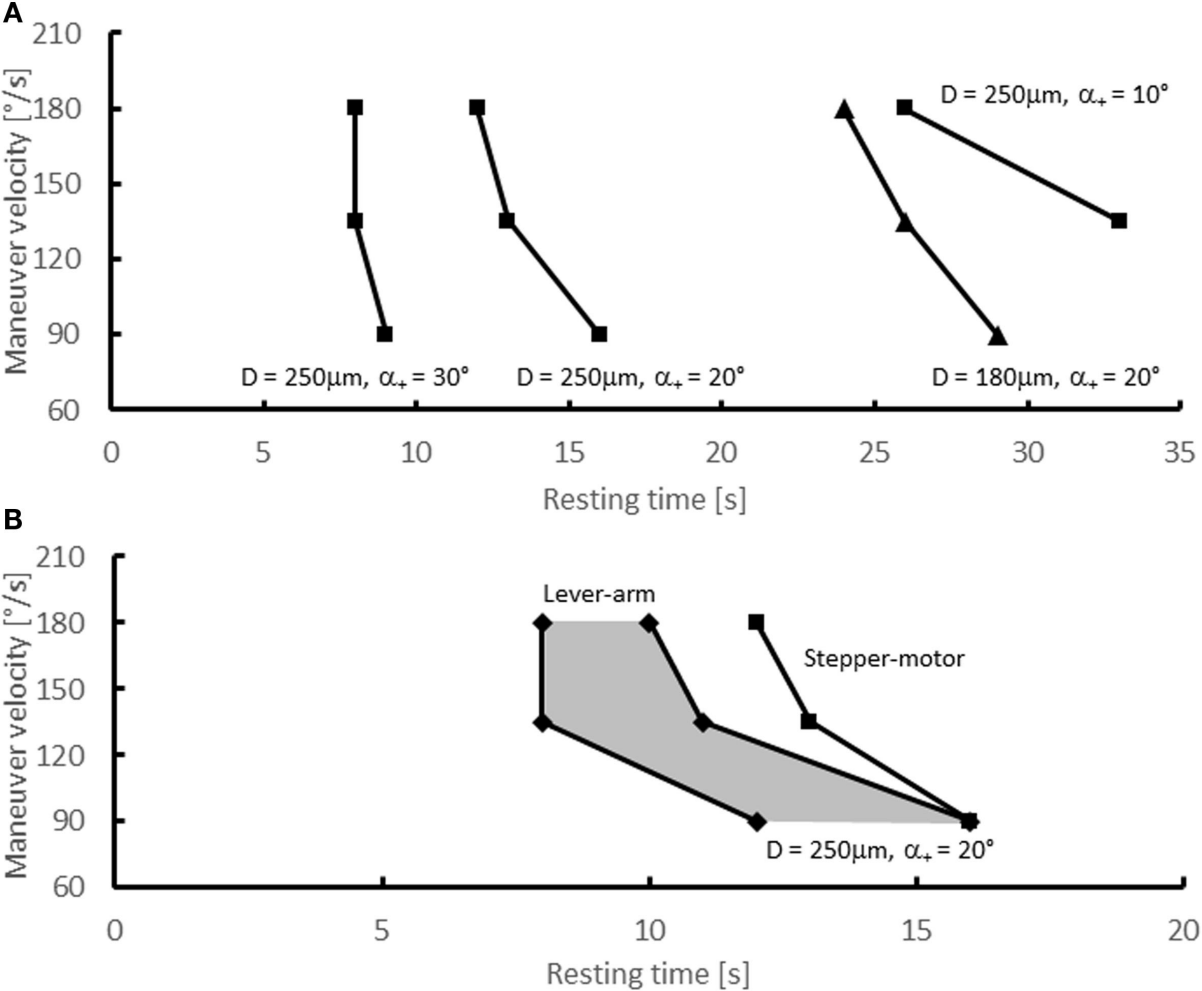
**Experimental results – critical resting times**. Critical resting times as function of the maneuver velocity for various experimental configurations. **(A)** Stepper-motor experiments with *D* = 250 μm (•) and α_+_ = 10°, 20°, 30° and with *D* = 180 μm (▴) and α_+_ = 20°; **(B)** lever-arm experiments (■) with *D* = 250 μm and α_+_ = 20°. The shaded area indicates the range of resting times for which the lever-arm experiments did not yield conclusive results. For comparison, the critical resting times from the stepper-motor experiments for *D* = 250 μm (•) and α_+_ = 20°are shown as well [identical to data shown in **(A)**].

For α_+_ = 10° and *v* = 90°/s, a successful canalith repositioning was also not possible. The maneuver was successful only for maneuver velocities of 135 and 180°/s, if the resting time was at least 33 and 26 s, respectively. For an extension angle of 20°, resting times of at least 16 s were required for a successful repositioning of the particle. Higher maneuver velocities required somewhat shorter resting times. For α_+_ = 30°, the SM was successful for *T*_p_ even below 10 s, and the maneuver velocity had only a minor effect on the critical time. The resulting critical resting times are summarized in Table 2 and in Figure 5A.

### Variation of Particle Size

To assess the effect of the particle size on the outcome of the SM, the stepper-motor experiments were repeated for α_+_ = 20° with a smaller particle size *D* = 180 μm (Figure 5A and Table 2). The critical times *T*_p_ (29 s for 90°/s, 26 s for 135°/s, 24 s for 180°/s) were approximately twice as high as the critical times for the large particle.

### Lever-Arm Experiments

Experiments with the lever-arm setup were carried out to assess the effect of the shifted pivot on the canalith repositioning. Figure 5B and Table 2 present the results for a particle of 250 μm diameter and an extension angle of α_+_ = 20°. For a certain range of resting times, the results were inconclusive, i.e., in some instances the maneuver was successful, but in other instances it failed. This can be attributed to small variations in the manually performed maneuvers.

## Discussion

The major findings of this *in vitro* study on the theoretically relevant parameters for SM are the following. First, a sufficiently long resting time between the two movements is a primary determinant for successful SMs. Second, it is advantageous to extend the movement of the head beyond the earth horizontal by 20° (so-called Sémont+); if the SM is only performed to the earth horizontal, the otoconia are unlikely to be repositioned successfully. Third, the angular velocity has only a minor effect on the success of the SM in the tested range (90, 135, 180°/s).

Basing clinical recommendations on the experimental results would seem likely to improve the effectiveness of the SM. However, it is necessary to consider the limitations of the present experiments. First, we have to assess the differences between the SM performed in the stepper-motor experiments and an actual SM performed on a patient. To this end, we compared stepper-motor results with results from the lever-arm experiments, which closely modeled the actual clinical situation. Second, the particle size has an effect on the success of the maneuver. This is a critical point because the canalith size is not known for individual patients. In the following paragraphs, we discuss both points in detail before formulating clinical recommendations.

The experiments with the lever-arm setup showed that the critical times between the steps are slightly less than for corresponding configurations with the stepper-motor experiment. This indicates that centripetal accelerations that are only present in the lever-arm setup are beneficial for canalith repositioning. Therefore, the critical resting times in clinical practice are probably less than in the stepper-motor experiments. However, the difference in the critical times between lever-arm and stepper-motor setup is 2 s or less, which is quite small given that other uncertainties (e.g., unknown particle size) can lead to greater differences.

After the first movement by 90° + α_+_, the particles in our experiment settled at the lowest point of the SCC after approximately 10–30 s depending on maneuver velocity, extension angle, and particle size. For comparison, experiments (29) with glass beads (15–25 μm diameter) in SCC of Opsanus Tau (inclined by 21°) indicated that the beads were moving at velocities of up to 80 μm/s and that they settled after 62–65 s. Furthermore, theoretical and computational studies indicate that the settling speed scales with the inverse of the particle cross-section (22, 24, 30). According to this theory, small particles (180 μm) require nearly twice as long to settle as large particles (250 μm) because the ratio of their respective cross-sections is nearly two: π ⋅ (250 μm)^2^/π ⋅ (180 μm)^2^ ≈ 1.9. This agrees quite well with the doubling of critical times illustrated in Figure 5A.

Human utricular otoconia are reported to have diameters ranging from 1 to 30 μm (31, 32). Canaliths are thought to consist of larger lumps of multiple otoconia (19) as well as of single otoconia, which may detach from the otolith macula due to structural degeneration (33), drugs (34), and mechanical insult (23). Experiments in animal models (35) suggested that debris moving inside an SCC stimulates ampulla receptors only if the particles are of a suitable size (microsphere diameter exceeded about 50 μm). Theoretical and numerical studies predict a positional nystagmus of only 2°/s for a canalith with a diameter of 15 μm, 5°/s for a 30-μm canalith (22), and a nystagmus of approximately 20°/s for a canalith with a diameter of 57 μm (36). These results suggest that single otoconia of 15 μm or less are clinically not very relevant targets for canalith repositioning with the SM. For our further discussion, we postulate that the SM should target otoconia with a diameter of at least 25 μm. Such otoconia would correspond to 125 μm-particles in our experiment. Their cross-section is 4 times smaller than for 250 μm-particles, with the result that the critical times are expected to be 4 times larger than the critical times for the 250 μm-particles reported in Table 2.

Finally, it should be kept in mind that particles in the SCC model settle approximately 20% more slowly than in a human SCC. Therefore, the predicted critical times can be considered conservative estimates.

On the basis of the present results and discussion, we propose the following clinical recommendations to improve the success rate of the SM.

First, the time between the two movements should be at least 45 s; second, the measured angle for the first step of the SM should ideally be 110° (not only 90° as had been recommended) and for the second step 220° (not only 180°). If a patient tolerates an extension angle of 30° beyond the horizontal, the time between the steps can be reduced. Third, the SM should be performed with an angular velocity of around 135°/s (i.e., about 0.66 s for the 90° movement and about 1.33 s for the 180° movement). Successful SMs can also be performed at lower velocities (90°/s), which is of particular interest in immobile or obese patients, provided that the resting time is increased to at least 60 s.

These recommendations are based on the *in vitro* model with single spherical particles. The effect of multiple, non-spherical particles and of possible lumps of particles has not yet been considered. Nevertheless, the present results could have a considerable impact on daily clinical practice. Since the time between the movement and the body position are easy to measure, this could be the theoretical basis of controlled clinical trials to increase the efficacy of the treatment of the most frequent cause of vertigo. Further, these findings could provide the basis for a clinical trial comparing Sémont+ with the regular SM in terms of their efficacy.

## Author Contributions

DO – study concept and design, analysis and interpretation of data, writing and critical revision of the manuscript for intellectual content, and study supervision; AN and EZ – acquisition of data, analysis and interpretation of data, critical revision of the manuscript for intellectual content; RK – analysis and interpretation of data, writing, and critical revision of the manuscript for intellectual content; GM – study concept and design, analysis and interpretation of data, writing and critical revision of the manuscript for intellectual content; MS – idea of the study, study concept and design, analysis and interpretation of data, writing, and critical revision of the manuscript for intellectual content.

## Conflict of Interest Statement

MS is Joint Chief Editor of the Journal of Neurology, Editor in Chief of Frontiers of Neuro-otology, and Section Editor of F1000. He has received speaker’s honoraria from Abbott, Actelion, Biogen, Eisai, GSK, Henning Pharma, Interacoustics, MSD, Otometrics, Pierre-Fabre, TEVA, and UCB. He acts as a consultant for Abbott, Actelion, Heel, and Sensorion. The remaining authors declare that the research was conducted in the absence of any commercial or financial relationships that could be construed as a potential conflict of interest.
